# Diagnosing complications following cochlear implantation using transcutaneous ultrasound

**DOI:** 10.1007/s00405-021-07128-2

**Published:** 2021-10-26

**Authors:** Robin Rupp, Vivian Thimsen, Matthias Balk, Sarina K. Mueller, Matti Sievert, Konstantinos Mantsopoulos, Ulrich Hoppe, Joachim Hornung, Heinrich Iro, Antoniu-Oreste Gostian

**Affiliations:** Medical Faculty, Department of Otorhinolaryngology, Head and Neck Surgery, Friedrich-Alexander-Universität Erlangen-Nürnberg (FAU), Waldstraße 1, 91054 Erlangen, Germany

**Keywords:** Cochlear implant, Transcutaneous ultrasound, Diagnostic sonography, Magnet dislocation, Haematoma, Seroma

## Abstract

**Purpose:**

The aim of this study was to investigate the feasibility and reliability of transcutaneous ultrasound for the detection of complications after cochlear implantation.

**Methods:**

In a single center retrospective cohort study, 115 consecutive cases of suspected complications after cochlear implantation (intervention group) were examined. The rate of pathologic ultrasound findings for specific leading symptoms and diagnoses was compared to a control group comprising twenty consecutive cochlear implants in symptom-free patients.

**Results:**

Diagnostic ultrasound showed distinctly more pathologic findings in the intervention group (*n* = 67; 58.3%; *p* < 0.001) compared to the control group (*n* = 1; 5%). Ultrasound revealed significantly more pathologic findings in haematoma or seroma around the implant (*n* = 17; 100%; *p* < 0.001; *ϕ* = 0.94) and magnet dislocation (*n* = 44; 97.7%; *p* < 0.001; *ϕ* = 0.92) confirmed by a strong effect. Ultrasound examination showed a medium to high effect size in patients presenting with local infections (*n* = 3; 21.4%; *p* = 0.283; *ϕ* = 0.25) and skin flap oedema (*n* = 2; 50%; *p* = 0.061; *ϕ* = 0.51). In contrast, ultrasound examinations displayed a low effect size in undefined cephalgia (0%; *p* = 0.444; *ϕ* = 0.17) and device malfunction or failure (0%; *p* > 0.999; *ϕ* = 0.13).

**Conclusion:**

Transcutaneous ultrasound can be advocated as a feasible and effective method in the diagnostic work-up of magnet dislocation and haematoma or seroma around the implant following cochlear implantation. Contrary, ultrasound findings can be expected to be inconspicuous in patients presenting with undefined cephalgia and device malfunction or failure.

## Introduction

Cochlear implantation has proved to be an effective and safe therapy for patients with moderate to severe hearing loss [1, 2]. Nevertheless, complications relating to the implant occur in 3.7–12.8% of implanted patients [3–5]. Especially in children, trauma is a major factor for complications [6]. The application of external magnetic fields, e.g., magnetic resonance imaging (MRI), may also cause complications, such as magnet dislocation [7, 8]. In general, complications are classified into minor or major [5, 9]. Minor complications can usually be treated conservatively, whereas major complications require hospitalisation of the patient or additional surgery, including device explantation. Although many studies have focused on the surgical and medical complications that occur after cochlear implantation [4–6, 10], data on imaging methods for the optimal diagnostic workup of these complications is limited and inconclusive. Mostly, temporal bone computed tomography (CT) or X-ray examination is recommended when dealing with cochlear implant (CI) complications [7, 8, 11–14]. In this context, 82.4% of CT scans showed no abnormalities in CI complications but exposed the patients to radiation [15]. In contrast, transcutaneous ultrasound completely avoids radiation exposure. This imaging modality has been described in the diagnosis of haematoma around the CI and dislocation of the internal CI magnet [16–18]. However, data on the use of ultrasound in other complications are rare and consist of case reports only [13, 19, 20]. Therefore, the objective of this retrospective study was to investigate whether ultrasound is a helpful and reliable tool in the detection of complications after cochlear implantation.

## Materials and methods

### Patients and examinations

This retrospective study was conducted at a tertiary referral medical centre (Department of Otorhinolaryngology and Head and Neck Surgery, Friedrich-Alexander-Universität Erlangen-Nürnberg (FAU), Erlangen, Germany) and was approved by the institutional review board (application numbers: 229_20 B and 99_21 Bc). The requirement of informed consent from patients with CI complications was waived as images acquired for clinical diagnostic purposes were reviewed retrospectively.

Patients with an ultrasound examination in the diagnostic workup presenting with a suspected complication after cochlear implantation between January 1st, 2006 and December 31st, 2020 were eligible for the study and are defined as the intervention group. Inclusion criteria were as follows: CI present; clinical suspicion of complication defined by the following symptoms: swelling or pain around the CI, local skin reddening or atrophy over the CI, reduced CI performance; transcutaneous ultrasound examination during the diagnostic workup, complete medical record. The most prominent symptom was defined as the leading symptom. The following exclusion criteria applied: incomplete medical record, missing ultrasound during the diagnostic workup and refusal to undergo ultrasound examination.

20 consecutive CIs of 19 patients without any symptoms were examined by ultrasound and defined as the control group. Informed consent was obtained from each patient in the control group. For ethical reasons, children were not included in the control group.

The diagnostic workup of all patients comprised a thorough clinical examination followed by an ultrasound examination (Sonoline Elegra with 7.5 MHz transducer; Acuson S2000™ with linear transducer 9L4 (4–9 MHz) and 14L5 (5–14 MHz); Acuson Sequoia™ with linear transducer 10L4 (2.9–9.9 MHz) and 18L6 (4.6–17.8 MHz); Siemens Healthineers AG, Erlangen, Germany) by an ENT specialist. In case of suspected CI malfunction or failure, the device was checked technically. If the diagnosis was inconclusive, further imaging was performed, i.e., X-ray, CT-scan or digital volume tomography (DVT). Any hypoechoic or anechoic structure adjacent to the implant signifying the presence of fluid, a tilted or completely dislocated internal magnet or tissue swelling around the implant was classified as “pathological”. Reports with completely unremarkable findings were classified as “normal”.

### Statistical analysis

Metric variables are presented as mean values ± 1 standard deviation (SD), minimum (min.) and maximum (max.). Statistical calculations were performed using SPSS (IBM SPSS Statistics 22.0, IBM, New York, NY). The Chi-square test was used for the comparison of nominal variables. If the reported frequency of a pathological ultrasound was below 5, Fisher’s exact test was conducted. The Shapiro–Wilk test was performed to test for normal distribution. In cases, where metric variables were not normally distributed, the Mann–Whitney*U* test was used. A *p*value ≤ 0.05 was considered as statistically significant. Correction for multiple testing was performed using the Bonferroni correction.

For nominal variables, the effect size *ϕ* was calculated on a post-hoc level, with *ϕ* = 0.1 displaying a small effect, *ϕ* = 0.3 representing a medium and *ϕ* = 0.5 a strong effect.

## Results

### Patient characteristics

During the study period, a total of 102 patients, accounting for 115 cases of suspected CI complications in 105 different cochlear implants (49 right-sided implants), underwent ultrasound examination during the diagnostic workup. 15 out of these 105 implants (14.3%) had been implanted at an external clinic. In four cases (3.5%), additional imaging modalities were applied: one X-ray, two CT scans and one DVT.

The 102 included patients (59 ♀; 35 with bilateral implants) averaged 45.5 ± 26.1 years (yr) (min. 0.3 yr, max. 92.6 yr) at the time of initial ultrasound examination. The mean time from implantation to CI complication was 4.2 ± 4.7 yr (min. 1 day, max. 27.1 yr). 23 patients (22.5%) accounting for 26 cases (22.6%) were younger than 18 years of age (3.7 ± 3.5 yr). All but one patient developed complications more than 1 week after cochlear implantation. One out of 35 patients who were implanted bilaterally showed complications on both sides. Two patients with CI complications had undergone re-implantation and developed complications with their new implant after 7 months and 5.5 yr, respectively.

In the control group, 20 devices were examined in 19 patients (9 ♀; one with bilateral implants). As for ethical reasons only adult patients were included in the control group, these patients were significantly older than the patients in the intervention group at the time of initial ultrasound examination (62.3 ± 13.5 yr; min. 41.7 yr; max. 84.6 yr; *Z* =  − 2.618; *p* = 0.008). The mean time from implantation to CI complication was comparable to the intervention group (4.2 ± 3.9 yr; min. 33 days; max. 15.7 yr; *Z* =  − 0.426; *p* = 0.674).

The intervention group included 95 devices (90.5%) from Cochlear® (Cochlear Limited, Sydney, Australia; CI24M = 6; CI24R (CS) = 1; CI24RE (CA) = 35; CI512 = 34; CI532 = 17; CI612 = 1; CI632 = 1) and 7 implants (6.7%) from MED-EL® (MED-EL, Innsbruck, Austria; PULSAR CI-100 = 1; Concerto Flex 28 = 3; Synchrony Flex 28 = 3). Three patients (2.8%) had been implanted with implants from Advanced Bionics® (Advanced Bionics, Valencia, United States; HiRes 90 k = 1; HiRes Ultra = 1; HiRes Ultra 3D = 1).

The control group included 13 devices (65%) from Cochlear® (CI24RE (CA) = 1; CI512 = 4; CI532 = 2; CI612 = 1; CI632 = 5) and 7 implants (35%) from MED-EL® (PULSAR CI-100 = 2; Concerto Flex 28 = 1; Synchrony Flex 28 = 4).

In total, in 49 of 115 cases (42.6%) patients reported having had an MRI examination before the onset of symptoms; 17 cases (14.8%) were due to a trauma. The most frequent leading symptom was swelling around the implant (60 cases; 52.2%). 18 cases (15.6%) showed local skin reactions with skin reddening and atrophy or ulcer formation over the receiver/stimulator (RS). Pain was the leading symptom in 26 (22.6%) cases. Reduced CI performance was the chief complaint in 11 (9.5%) cases.

**Fig. 1:**
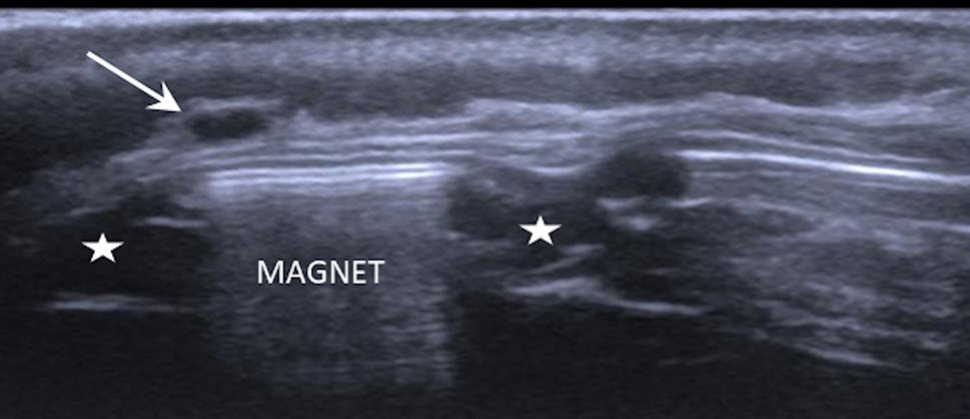
Anechoic area above a CI612 diagnosed as a minimal postoperative seroma 4 weeks after cochlear implantation in a patient without any symptoms. The anechoic magnet housing is indicated by asterisks; the hyperechoic magnet is marked as “MAGNET”; the arrow indicates the seroma that was measured at 1.2 × 3.6 mm

In the control group, one out of 20 ultrasound examinations showed pathological findings (5%). In this single case, a very small anechoic area diagnosed as postoperative seroma was detected above the internal magnet in a patient 4 weeks after implantation, as shown in Fig. 1.

### Pathological findings for leading symptoms

With 67 out of 115 examinations (58.3%), ultrasound showed significantly more pathological findings in the intervention group than in the control group (χ^2^_(1)_ = 19.33; *p* < 0.001; *ϕ* = 0.38). Compared to the control group, ultrasound detected significantly more pathological findings with swelling as the leading symptom associated with a high effect size (*n* = 50; 83.3%; χ^2^_(1)_ = 39.83; *p* < 0.001; *ϕ* = 0.70) as well as cases with local skin reactions (*n* = 11; 61.1%; χ^2^_(1)_ = 13.81; *p* < 0.001; *ϕ* = 0.60). In the case of pain as the leading symptom, ultrasound examination did not reveal an increased incidence of pathological findings showing a small effect, with 5 out of 26 examinations showing abnormalities (19.2%; *p* = 0.212, *ϕ* = 0.21). Likewise, in patients who presented with reduced CI performance, pathological findings were not distinctly increased, with one out of 11 ultrasound examinations showing abnormalities (9.1%; *p* > 0.999; *ϕ* = 0.08). Table 1.A shows pathological ultrasound examinations for leading symptoms in the intervention group compared with the control group.

**Table 1:** Pathological ultrasound examinations for leading symptoms and for specific diagnosis: intervention group versus control group

	Intervention group (*n*)	Pathological ultrasound findings (*n*/%)	*p* value	Effect size *ϕ*
(A) Leading symptom				
Swelling	60	50 (83.3%)	< 0.001^*^	0.70
Local skin reaction	18	11 (61.1%)	< 0.001^*^	0.60
Pain	26	5 (19.2%)	0.212	0.21
Reduced CI performance	11	1 (9.1%)	> 0.999	0.08
∑	115	67 (58.3%)		
(B) Diagnosis				
Haematoma/Seroma	17	17 (100%)	< 0.001*	0.94
Magnet dislocation	45	44 (97.7%)	< 0.001*	0.92
Local infection	14	3 (21.4%)	0.283	0.25
Skin flap oedema	4	2 (50%)	0.061	0.51
Device malfunction/failure	9	0 (0%)	> 0.999	0.13
Undefined cephalgia	25	0 (0%)	0.444	0.17
Trichilemmal cyst	1	1 (100%)	*a*	*a*
∑	115	67 (58.3%)		

*CI* cochlear implant

^*^ Indicates a significant difference compared with the control group

^*a*^ No test performed due to small number of the respective diagnosis

### Pathological findings for a specific diagnosis

**Fig. 2:**
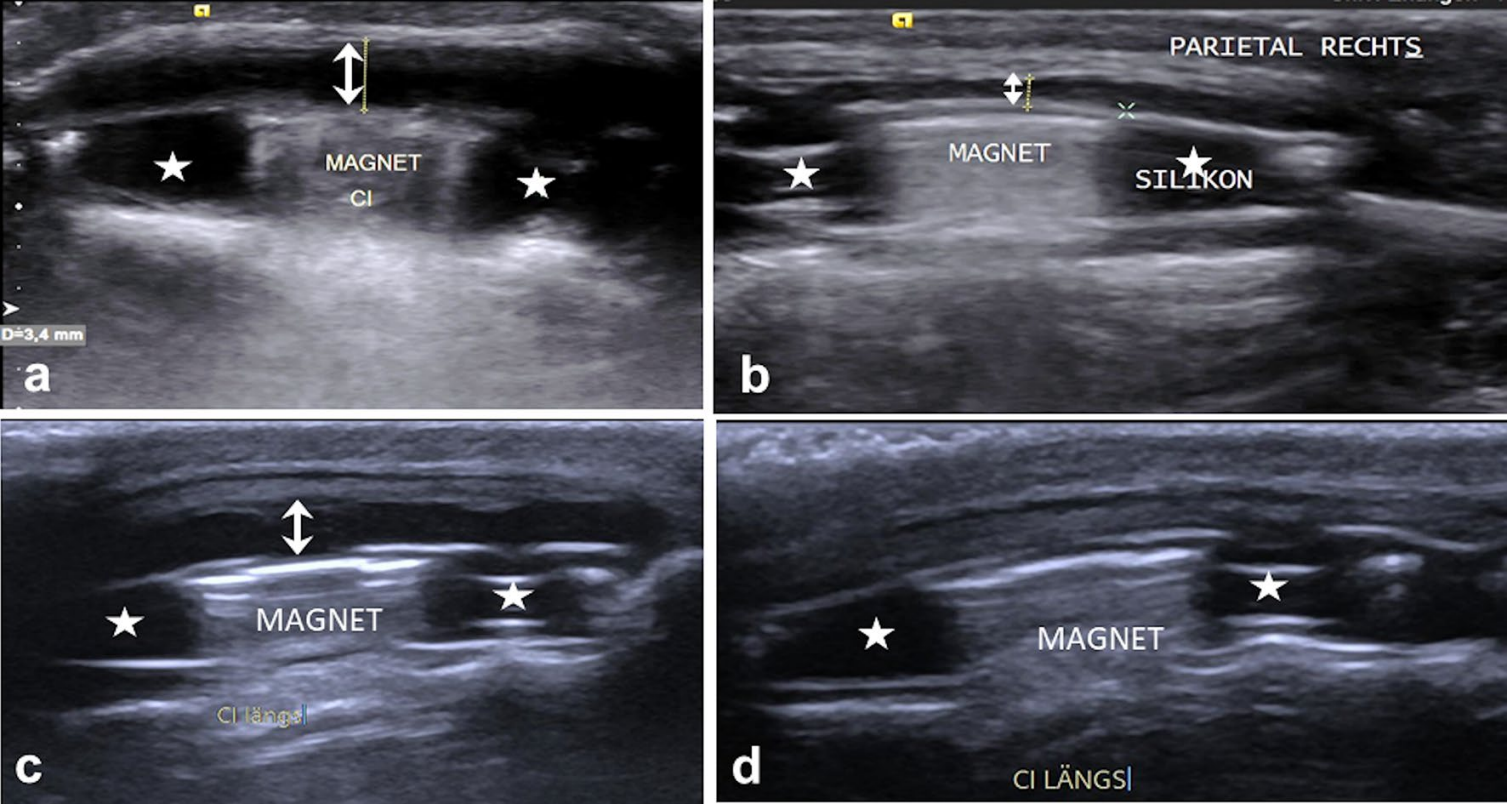
Patients with haematoma and seroma around their CI marked by double arrows. The anechoic magnet housing is indicated by asterisks; the implant magnet is indicated by “MAGNET”. **a** 4-year-old child implanted with a CI24RE (CA) on the right side diagnosed with haematoma around the CI after trauma; an anechoic area over the implant is measured at 3.4 mm in height; the haematoma was aspirated and a pressure bandage and oral antibiotics were administered. **b** Follow-up ultrasound showed successful reduction of the haematoma over the implant to 1.3 mm 9 days later. **c** A 16-year-old patient with a CI24RE (CA) on the right side diagnosed with a seroma around the device that was measured at 2.5 mm in height. The patient was advised not to use his device temporarily. **d** Follow-up ultrasound 17 days later showed no seroma

In 17 cases (14.8%) with suspicion of haematoma or seroma based on the clinical examination, all ultrasound examinations performed showed pathological findings represented by a strong effect (χ^2^_(1)_ = 33.19; *p* < 0.001; *ϕ* = 0.94). Five out of these 17 patients (28.4%) had a history of trauma. Conservative treatment was successful in 14 out of 17 cases. Surgical therapy (*n* = 3) included removal of haematoma (*n* = 1) and seroma (*n* = 1) as well as explantation of the device (*n* = 1). Figure 2 shows the photo documentation and follow-up examination of a haematoma as well as a seroma around the CI.

The internal CI magnet was dislocated in 45 out of 115 cases (39.1%). Ultrasound examination showed abnormalities in all but one of these cases (97.7%) represented by a strong effect size (χ^2^_(1)_ = 55.95; *p* < 0.001, *ϕ* = 0.92). Additional DVT was performed in one case to rule out electrode dislocation. All but two cases of magnet dislocation occurred after an MRI examination. 31 magnets were repositioned surgically. In 13 cases with only partial dislocation of the magnet, i.e., canting of the magnet, a manual magnet repositioning manoeuvre was successfully performed. One patient was scheduled for surgical magnet repositioning but experienced a spontaneous repositioning of the magnet.

Ultrasound examination was abnormal in three out of 14 cases that were diagnosed with a local infection in the area of the implant, representing a medium effect size (21.4%; Fisher’s *Z*: *p* = 0.283; *ϕ* = 0.25). Four of these 14 cases had a history of trauma. All patients received conservative treatment, but one device had to be explanted in the course of time.

In nine cases (7.8%), device malfunction (*n* = 7; 6.1%) and device failure (*n* = 2; 1.7%) was diagnosed after a technical check-up, while ultrasound examination revealed normal findings in all nine cases, representing a small effect (Fisher’s *Z*: *p* > 0.999; *ϕ* = 0.13). An additional CT scan that was performed in one case revealed no pathological findings, and the device had to be replaced in the course of time. The two cases of device failure occurred after a head trauma and the implants had to be replaced.

In 25 cases (21.7%), undefined cephalgia was diagnosed based on the clinical examination. Subsequent ultrasound examination revealed no abnormalities, representing a small effect size (Fisher’s *Z*: *p* = 0.444; *ϕ* = 0.17). One patient underwent a CT scan as additional imaging that showed normal findings. In the course of time, three devices had to be explanted due to chronic pain in the implant area.

In four out of 115 cases (3.5%), skin flap oedema over the RS was the leading diagnosis. Two out of all four ultrasound examinations (50%) showed abnormalities, i.e., increased height of the tissue overlying the implant, representing a high effect size (Fisher’s *Z*: *p* = 0.061; *ϕ* = 0.51). No additional imaging was performed. In three cases, the skin flap thickness decreased over time as the patients were still in the early postoperative period. In one case, the skin flap was thinned successfully in local anaesthesia.

In one case, a cyst over the RS was diagnosed using ultrasound. The patient received an X-ray examination in Stenvers view as additional imaging to rule out dislocation of the internal magnet. The cyst could be removed completely in general anaesthesia and was confirmed as a trichilemmal cyst by the pathologist.

**Fig. 3:**
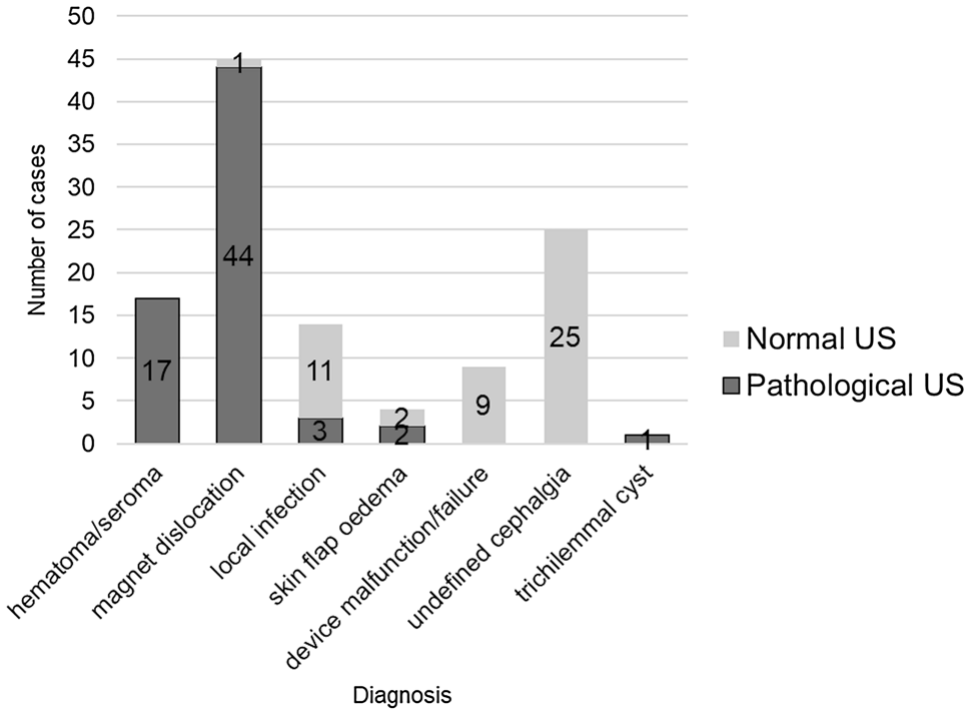
Proportion of pathological ultrasound findings for specific diagnoses. *US* ultrasound

Table 1.B shows pathological ultrasound examinations for specific diagnosis in the intervention group compared with the control group. Figure 3 presents the proportion of pathological ultrasound examinations for specific diagnosis.

## Discussion

The present study demonstrates that transcutaneous ultrasound examination is a feasible and reliable diagnostic tool in patients with complications after cochlear implantation presenting with swelling and local skin reactions in the region of their implant. Pathological findings with ultrasound can be expected in most cases of magnet dislocation and haematoma or seroma, as well as in numerous cases of local infection or skin flap oedema. However, ultrasound findings can be expected to be unremarkable in patients presenting with undefined cephalgia and device malfunction or failure.

Complication rates after cochlear implantation are low [1, 5]. Nevertheless, in the worst case, infection of the implant or chronic pain can lead to a necessity for device explantation [10, 13]. For this reason, any sign of a complication requires immediate and thorough diagnosis and therapy. CT scans and X-rays have both been recommended for various CI complications. Recently, CT scans were evaluated regarding the diagnosis of delayed complications after cochlear implantation and reported that 82.4% of CT scans showed no abnormalities. Still, an abnormal finding correlated significantly with a necessity for surgical therapy [15]. Similarly, CT scans and X-rays have been described for evaluating atypical pain in the device area in children and adults [13, 21]. Furthermore, X-rays are widely used in diagnosing magnet dislocation [7, 8, 11]. However, Epperson et al. [22] report a case, where magnet dislocation was initially overseen on X-ray. They state that minimal literature is currently available on the imaging of magnet dislocation. Holtmann et al. [14] found that by diagnosing magnet dislocation using CT, prompt diagnosis was delayed in some cases because of artefacts. Nevertheless, CT has proved its worth in rare but serious CI complications, such as subdural and epidural haematoma [23–25]. However, it must be borne in mind that CTs and X-rays should be indicated with restraint due to the unavoidable radiation dose. This applies all the more to children.

MRI that avoids radiation exposure is not approved in older cochlear implants [26]. Even in the latest CIs that are MRI-compatible, the magnet of the RS and the implant itself produce an artifact that impedes the proper assessment of the implant area [27]. In this regard, transcutaneous ultrasound is a valuable imaging modality that avoids radiation exposure and has been described for the diagnostic workup of haematoma around the CI and for detecting magnet dislocation [16–18]. However, the literature on the feasibility and reliability of ultrasound in other CI complications has so far been limited to a few case reports [13, 19, 20].

Recently, Wolber et al. [15] recommended performing a CT scan in any case of delayed CI complication. According to our results presented in this study, we would propose altering the diagnostic workup for these patients, depending on the experience in ultrasound examination at the respective department. Most patients presenting with swelling or local skin reactions at the area of the implant can be reliably diagnosed by transcutaneous ultrasound examination as displayed by a high effect size when compared with the control group. As shown in the present study, ultrasound examination was beneficial in showing a medium to high effect size when diagnosing haematoma or seroma, magnet dislocation, local infection and skin flap oedema. Accordingly, X-rays or CT scans should be regarded as diagnostic tools of second choice if ultrasound examination is inconclusive. This workup scheme has the advantage of potentially avoiding unnecessary radiation exposure. In contrast, when patients present initially with reduced CI performance as a sign of device malfunction or failure, ultrasound examination can be expected to show normal findings. Additional imaging by CT or X-ray should then be indicated on an individual basis, as Wolber et al. [15] report that only 10% of patients with device failure showed an abnormal CT scan but surgery had to be performed in all cases. Apart from imaging, a thorough technical check-up is essential. Due to inevitable artefacts that strongly impair image assessment, MRI is not recommended in CI complications.

At our department, ultrasound examination is readily available and is performed by the ENT specialist. By this means, the implant can be evaluated multidimensionally in a dynamic exploration. Furthermore, examination can be performed repeatedly as a post-therapeutic follow-up examination if required. However, due to its technical properties, ultrasound examination is limited by bony structures that completely reflect the ultrasonic waves. Therefore, if a CI complication exceeds the RS and the surrounding soft tissue, a temporal bone CT scan is inevitable.

The study is somewhat limited by the fact that only patients with an ultrasound examination of their CI were included, leading to the exclusion of patients with complications after cochlear implantation who were diagnosed by X-ray or CT only. Therefore, no direct comparison of these imaging modalities can be made, as this was beyond the scope of the study. Second, the retrospective character of this study needs to be taken into account when interpreting the results. In addition, the ultrasound examinations were performed by different examiners, so that inconsistent, examiner-dependent findings cannot be completely excluded. Still, the presented study represents the largest number of ultrasound examinations performed to date for the diagnosis of CI complications. The vast majority of implants in the study group came from one single manufacturer. In this context, it is important to note that devices from this manufacturer are used most often at our department. However, complication rates for each manufacturer were not calculated as this was beyond the scope of the study.

In conclusion, transcutaneous ultrasound examination can be advocated as a feasible and effective method in the diagnostic workup of CI complications clinically defined by swelling or local skin reactions around the CI. Ultrasound reliably shows pathological results in cases of magnet dislocation and haematoma or seroma around the implant. In contrast, the diagnostic value is limited in patients presenting with undefined cephalgia, device malfunction or device failure. Thus, diagnostic ultrasound can obviate the necessity of imaging by X-ray and CT, which can be additionally indicated if necessary.

## References

[1] Dazert S, Thomas JP, Loth A, Zahnert T, Stover T (2020). Cochlear implantation. Dtsch Arztebl Int.

[2] Hoppe U, Hocke T, Hast A, Iro H (2021). Cochlear implantation in candidates with moderate-to-severe hearing loss and poor speech perception. Laryngoscope.

[3] Carlson ML (2020). Cochlear implantation in adults. N Engl J Med.

[4] Terry B, Kelt RE, Jeyakumar A (2015). Delayed complications after cochlear implantation. JAMA Otolaryngol Head Neck Surg.

[5] Binnetoglu A, Demir B, Batman C (2020). Surgical complications of cochlear implantation: a 25-year retrospective analysis of cases in a tertiary academic center. Eur Arch Otorhinolaryngol.

[6] Loundon N, Blanchard M, Roger G, Denoyelle F, Garabedian EN (2010). Medical and surgical complications in pediatric cochlear implantation. Arch Otolaryngol Head Neck Surg.

[7] Grupe G, Wagner J, Hofmann S, Stratmann A, Mittmann P, Ernst A, Todt I (2017). Prevalence and complications of MRI scans of cochlear implant patients: English version. HNO.

[8] Fussell WL, Patel NS, Carlson ML, Neff BA, Watson RE, Lane JI, Driscoll CLW (2021). Cochlear implants and magnetic resonance imaging: experience with over 100 studies performed with magnets in place. Otol Neurotol.

[9] Hoffman RA, Cohen NL (1995). Complications of cochlear implant surgery. Ann Otol Rhinol Laryngol Suppl.

[10] Layfield E, Hwa TP, Naples J, Maina I, Brant JA, Eliades SJ, Bigelow DC, Ruckenstein MJ (2021). Failure and revision surgery after cochlear implantation in the adult population: a 10-year single-institution retrospective and systematic review of the literature. Otol Neurotol.

[11] Nospes S, Brockmann MA, Lassig A (2019). MRI in patients with auditory implants equipped with implanted magnets-an update: overview and procedural management. Radiologe.

[12] Leinung M, Loth A, Groger M, Burck I, Vogl T, Stover T, Helbig S (2020). Cochlear implant magnet dislocation after MRI: surgical management and outcome. Eur Arch Otorhinolaryngol.

[13] Celerier C, Rouillon I, Blanchard M, Parodi M, Denoyelle F, Loundon N (2017). Pain after cochlear implantation: an unusual complication?. Otol Neurotol.

[14] Holtmann L, Hans S, Kaster F, Muller V, Lang S, Goricke S, Lang-Roth R, Arweiler-Harbeck D (2021). Magnet dislocation following magnetic resonance imaging in cochlear implant users: diagnostic pathways and managment. Cochlear Implants Int.

[15] Wolber P, Shabli S, Anagiotos A, Moellenhoff K, Schwarz D, Lang-Roth R (2021). The diagnostic value of computed tomography in delayed complications after cochlear implantation. Acta Otolaryngol.

[16] Filipo R, D'Elia C, Covelli E, Bertoli GA, De Seta E, Manganaro F, Mancini P (2010). Haematoma after cochlear implantation: management of a minor complication. Acta Otolaryngol.

[17] Rupp R, Hornung J, Balk M, Hoppe U, Iro H, Gostian AO (2020). Ultrasound in diagnosis of magnet dislocation of cochlear implants: a retrospective study in patients with surgical magnet repositioning and preinterventional ultrasound examination. Otol Neurotol.

[18] Rupp R, Hornung J, Balk M, Traxdorf M, Sievert M, Hoppe U, Iro H, Gostian AO (2021). Ultrasound-controlled manual magnet repositioning in magnet dislocation of cochlear implants. Otol Neurotol.

[19] Sakaida H, Usui S, Matsuda Y, Masuda S, Takeuchi K (2017). Sonographic diagnosis of acute mastoiditis and subsequent retroauricular abscess in a pediatric cochlear implant recipient: a case report. J Clin Ultrasound.

[20] Catli T, Olgun Y, Celik C, Gur H, Bayrak F, Olgun L (2015). Swelling around the implant body: a late complication of cochlear implantation. How to deal?. Cochlear Implants Int.

[21] Shapira Y, Yaar-Soffer Y, Hildesheimer M, Migirov L, Henkin Y (2015). Pain in cochlear implant recipients: an uncommon, yet serious, consequence of cochlear implantation. Laryngoscope.

[22] Epperson MV, Born HL, Greinwald J (2019). Radiologic recognition of cochlear implant magnet displacement. Int J Pediatr Otorhinolaryngol.

[23] Gosepath J, Maurer J, Mann WJ (2005). Epidural hematoma after cochlear implantation in a 2.5-year-old boy. Otol Neurotol.

[24] Li S, Qin Z, Zhang F, Li L, Qi S, Liu L (2014). Early complications following cochlear implantation in children and their management. Int J Pediatr Otorhinolaryngol.

[25] Gurbuz MS, Orakdogen M, Berkman MZ, Yuksel MO (2013). Subdural haematoma as a rare complication of cochlear implantation: case report and literature review. J Laryngol Otol.

[26] Srinivasan R, So CW, Amin N, Jaikaransingh D, D'Arco F, Nash R (2019). A review of the safety of MRI in cochlear implant patients with retained magnets. Clin Radiol.

[27] Sharon JD, Northcutt BG, Aygun N, Francis HW (2016). Magnetic resonance imaging at 1.5 tesla with a cochlear implant magnet in place: image quality and usability. Otol Neurotol.

